# Effect of Xingbi Gel Nasal Drops on Fyn-STAT5 Pathway in Nasal Mucosa Fibroblasts of Guinea Pigs with Allergic Rhinitis

**DOI:** 10.1155/2021/6686815

**Published:** 2021-03-20

**Authors:** Xiangli Zhuang, Bo Wu, Caixia Qiu, Si Ai, Jian Zheng

**Affiliations:** ^1^Academy of Integrative Medicine, Fujian University of Traditional Chinese Medicine, Fuzhou 350122, China; ^2^Second People's Hospital, Affiliated to Fujian University of Traditional Chinese Medicine, Fuzhou 350003, China; ^3^People's Hospital, Affiliated to Fujian University of Traditional Chinese Medicine, Fuzhou 350004, China; ^4^Institute of Pediatrics of Traditional Chinese Medicine, Fujian University of Traditional Chinese Medicine, Fuzhou 350122, China

## Abstract

Fyn-STAT5 is considered to be the frontier signaling pathway of IgE-mediated allergic reactions related to mast cell activation, but research on allergic rhinitis (AR) has been rarely reported. Xingbi gel nasal drops (XGND) are a compound preparation of traditional Chinese medicine, which has the exact therapeutic efficacy on AR. The current study aimed to observe the effects of XGND on Fyn-STAT5 pathway in AR guinea pig nasal mucosal fibroblasts *in vitro* and further illuminate the possible therapeutic mechanism of XGND on AR. The isolated and cultured nasal mucosa fibroblasts from AR guinea pigs were identified by immunocytochemical staining. Real-time PCR and western blot were performed to detect the mRNA and protein levels of the Fyn-STAT5 pathway and related cytokines in AR guinea pig nasal mucosal fibroblasts. The results indicated that XGND may interfere with the Fyn-STAT5 pathway by reducing the expression of Fyn and SCF and upregulating STAT5 and IL-10, thereby inhibiting proliferation and degranulation of mast cells, correcting Th1/Th2 immune imbalance, and then alleviating the immune response of AR fibroblasts. Our study revealed the possible regulatory mechanism of XGND in AR and laid an experimental foundation for improving the clinical efficacy of AR and enriching the clinical medication for AR.

## 1. Introduction

Allergic rhinitis (AR) refers to IgE-mediated noninfectious inflammatory diseases of the nasal mucosa that occur after exposure to allergens, which is characterized by nasal congestion, nasal discharge, sneezing, and nasal itching [1]. AR is one of the most common allergic diseases in the clinic. In the past 20 years, the incidence of AR has been increasing year by year due to environmental pollution, dietary habits, nutritional structure, and physical changes. At present, the incidence of AR has affected 10%∼25% of the global population [2], which is regarded as the “epidemic of the 21st century.” AR has a long course of disease, easy recurrence, and unstable curative efficacy, which brings a great economic burden and physical and mental distress to health resources and patients in many countries. The pathogenesis of AR is complex and is the result of multifactors and multilinks. Current studies suggest that the activation and degranulation of mast cells are the keys to the pathogenesis of AR. In allergic diseases, mast cells, located on the first line of defense for host exposure to allergens, are the core of immune activation [3]. IgE and its high affinity receptor Fc*ε*RI are the key mediators for mast cell activation, while tyrosine protein kinase Fyn is the key factor for activating the receptor Fc*ε*RI [4]. Signal transduction and activator of transcription 5 (STAT5) is an important member of the STAT family and plays a vital role in the development and survival of mast cells. Fyn-STAT5 is considered the frontier signaling pathway of IgE-mediated allergic reactions related to mast cell activation [5], but studies in AR are still rarely reported.

Traditional Chinese medicine (TCM) has obvious advantages in the treatment of AR in children. In recent years, it has achieved a good efficacy in treating AR and regulating immune function of the body [6, 7]. In another way, according to the mechanism of AR and the location of the lesion, the ideal approach for clinical treatment of AR is to use drugs on nasal mucosa directly to concentrate the distribution of drugs, produce the optimal efficacy, and minimize the systemic adverse reactions of drugs.

Xingbi gel nasal drops (XGND) represents an externally used patent medicine developed based on the clinical experience of Shoulin Huang, a famous doctor of TCM, which has a significant clinical efficacy on AR. In the present study, Fyn-STAT5 signaling pathway and related cytokines regulated by XGND in the nasal mucosa fibroblasts of AR guinea pigs were observed from the cellular and molecular perspective, to provide an experimental foundation for improving the clinical efficacy of AR and enriching clinical medication for AR.

## 2. Materials and Methods

### 2.1. Drugs

The prescription medicine of the XGND made by People's Hospital Affiliated to Fujian University of Traditional Chinese Medicine, including *Radix cynanchi Paniculati* (Pycnostelma paniculatum K. Schum) 100 g, *Periostracum Cicadae* (cicada slough) 45 g, *Calculus bovis Artifactus* (artificial bezoar) 12 g, and *Borneolum Syntheticum* (borneol) 2 g. All the herbs were decocted, filtered, and concentrated to a final dosage form of extract that has a crude drug concentration of 248 mg/ml. Budesonide nasal spray (brand name: Rhinocort) was purchased from AstraZeneca Pharmaceutical Co., Ltd., with a concentration of 1.28 mg/ml.

### 2.2. Animals

A total of 30 healthy and clean guinea pigs aged six weeks and weighing 200–250 g were ordered from the Shanghai Relaxation Experimental Animal Center under license number SCXK (Hu) 2014-0011. The environment and equipment for the experimental animals were provided by the Animal Laboratory of Fujian University of Traditional Chinese Medicine with the license number of SYXK (Min) 2014-0001. During the experiment, all animals were treated according to the “*Guiding Opinions on Treating Experimental Animals*” of the Ministry of Science and Technology of the People's Republic of China. The animal experiments have been reviewed and approved by the Ethics Committee of Fujian University of Traditional Chinese Medicine (no. FJTCM-2019-012).

### 2.3. Establishment of AR Model in Guinea Pigs

All guinea pigs were acclimated for seven days and divided into normal (*n* = 10) and model groups (*n* = 20) according to the random number table method. The model group was replicated into AR models using ovalbumin (OVA) (Sigma, USA) sensitization [8]. (1) Sensitization: each guinea pig was intraperitoneally injected with 1 ml suspension containing 0.5 mg/ml OVA + 30 mg/ml aluminum hydroxide (Sigma, USA), once every other day for seven times to make the guinea pig systemic sensitized; (2) challenge: on the 4th day after the completion of the sensitization, the guinea pigs were held up heads and challenged by dropping 50 *μ*l of 2% OVA solution into each nostril, once every other day for five times; (3) modeling effect evaluation according to the concentration of serum IgE (guinea pig IgE ELISA Kit, Shanghai Enzyme-linked, China) and National Allergic Rhinitis Diagnostic and Efficacy Evaluation Standards: within 30 minutes after each nasal challenge, the number of sneezes, nose scratching, and nasal discharge were observed and sneezing of 3∼9 times was scored as 1 point; 10∼14 times, 2 points; ≥15 times, 3 points; nose scratching of 2∼3 times was scored as 1 point; 4∼5 times, 2 points; ≥5 times, 3 points; runny nose to the nostrils was scored as 1 point, over the nostrils, 2 points; to all the faces, 3 points. A total score of ≥5 points, calculated by the superimposed method, indicated successful modeling.

### 2.4. Isolation and Culture of Primary Nasal Mucosa Fibroblasts

Both the healthy guinea pigs and AR guinea pigs were anesthetized and sacrificed. Under aseptic conditions, the nasal cavities were opened, and the nasal septa were removed to peel off the nasal mucosae on both sides. The sample was repeatedly washed with PBS containing 500 U/ml penicillin- streptomycin for 3∼5 times and cut into pieces of 1 mm^3^ in size and then digested with 0.1% type I collagenase (Sigma, USA) in a constant temperature water bath at 37°C for 4 h. The supernatant was collected and filtered through a 200-mesh cell sieve and then discarded after centrifuging at 1000 rpm/min for 5 min. The cells were resuspended in DMEM/F12 medium containing 10% fetal bovine serum (FBS) (Gibco, USA) and 1% penicillin-streptomycin, inoculated into a 25 cm^2^ cell culture flask, and cultured in a 5% CO_2_ incubator at 37°C. After 24 h, the cell culture medium was replaced for the first time to remove suspended impurities and cell debris, and then, the medium was changed every three days thereafter. The cells were subcultured (F1 generation) when they were grown to nearly full fusion into monolayer cells.

### 2.5. Purification of Fibroblasts by Differential Attachment

Fibroblasts were purified according to the different characteristics of adherent time between epithelial cells and fibroblasts. During cell passage, the single cell suspension was inoculated into a new flask and incubated for 15 to 20 min in an incubator. The cells were observed under the microscope, and the medium was gently decanted to remove the mixed cells that had not adhered. Finally, culture was carried out by adding a medium containing 10% FBS. Fibroblasts were identified by repeated purification up to the F3 generation.

### 2.6. Identification of Fibroblasts

#### 2.6.1. Morphological Observation

The morphology and growth of the cells were observed under an inverted phase contrast microscope.

#### 2.6.2. Identification by Immunocytochemistry

F3 generation cells of 1 × 10^5^/ml were inoculated in a six-well plate with glass coverslips. After the cells covered the cell slides by about 80%, the anti-vimentin antibody was stained by Strep Avidin-Biotin Complex (SABC) method, color was developed by DAB method, they were mildly counterstained with hematoxylin, and then the slides were observed under microscope followed by dehydration, clearing, and sealing with neutral gum. Cells without anti-vimentin antibody were used as negative control group.

### 2.7. Cell Counting Kit-8 (CCK-8) Assay

After successful identification, the F3 generation nasal mucosa fibroblasts of AR guinea pigs were inoculated into 96-well cell culture plates at a density of 5 × 10^4^/ml. Each group consisted of six duplicate wells; each well was added with 100 *μ*l of single cell suspension and cultured overnight in a 37°C incubator. When the cell growth coverage was about 60%, cells were treated with different concentrations of transforming growth factor-*β*1 (TGF-*β*1) (5, 10, 25, 50, and 100 ng/ml) (eBioscience, USA) for 6 h, 12 h, and 24 h and different concentrations of XGND (5, 10, 25, 50, and 100 *μ*g/ml) and Rhinocort (5, 10, 25, 50, and 100 *μ*g/ml) for 24 h, respectively. Then, the optical density (OD) value of each well was determined by CCK-8 assay (APExBIO, USA) at wavelength 450 nm. The experiment was repeated three times, and the results were averaged. The cell viability of the different groups was calculated by the cell viability rate of the NC group being 100% and then selected the concentration of the group with the optimal cell viability as the best condition for TGF-*β*1, XGND, and Rhinocort to interfere with fibroblasts.

### 2.8. Treatment of AR Fibroblasts

There were five cell groups studied including the normal group regarded as the nasal mucosa fibroblasts of healthy guinea pigs and four fibroblasts groups from AR guinea pigs: model group, TGF-*β*1 group, XGND group, and Rhinocort group. The fibroblasts of 5 × 10^4^/ml were in a medium containing 10% FBS in a flask. When the cell growth coverage was 60% to 70%, the medium was replaced with serum-free medium for 12 h, and then the normal group and model groups were cultured in 10% FBS medium, while the TGF-*β*1 group, XGND group, and Rhinocort group were cultured with the optimal concentration of TGF-*β*1, XGND and Rhinocort for 12 h, 24 h, and 48 h. The experiment was repeated three times.

### 2.9. Real-Time PCR

The mRNA was extracted from each group by Trizol (Life Technologies, USA). The concentrations of RNA and OD 260/280 were measured by ultramicro-nucleic acid analyzer. According to the instructions of the reverse transcription kit (TAKARA, Japan), a 20 *μ*l reverse transcription reaction system was prepared, and the reverse transcription conditions were 37°C for 15 min and 85°C for 5 sec. Then, with 1 *μ*l cDNA as template, a 20 *μ*l PCR reaction system was prepared (SYBR® Select Master Mix, Life Technologies, USA). The reaction conditions were as follows (7500 Fast Real-Time PCR System, Applied Biosystems, USA): predenaturation at 50°C for 2 min and 95°C for 2 min, followed by 40 cycles of denaturation at 95°C for 3 sec and annealing at 60°C for 30 sec. GAPDH was used as the internal control to calculate the 2^−ΔΔCt^ of relative expression levels of mRNAs of each gene. The experiment was repeated three times. The primer sequences of each gene are shown in Table 1.

### 2.10. Western Blot Analysis

The total protein from each group was extracted by RIPA lysate, and the concentration of each group was determined by BCA method (Beyotime, China). The SDS-PAGE gel electrophoresis was performed with 30 *μ*g of protein in each group. After electrophoresis, the target protein gel was cut off from the corresponding position referring to the prestained protein. The target protein was transferred to the PVDF membrane by semidry transfer method (Pierce™ 1-Step Transfer Buffer, Thermo, USA) and blocked at room temperature for 1∼2 h and then hybridized with rabbit anti-rat *β*-actin antibody (1 : 1000, CST, USA), rabbit anti-rat Fyn antibody (1 : 300, Santa Cruz, USA), and rabbit anti-rat STAT5 antibody (1 : 300, Santa Cruz, USA), and then incubated with goat anti-rabbit HRP-IgG antibody after washing for three times (1 : 5000, Bioss, China). The PVDF membranes were fully washed with TBST, and the images were developed by ECL chemiluminescence (ChemiDoc XRS + Imaging System, Bio-Rad, USA). The gray values of each band were read by Image Lab 4.0 software, and *β*-actin was used as an internal control to analyze the expression levels.

### 2.11. Statistical Analysis

The data were analyzed by SPSS 22.0 statistical software (IBM Corp., USA). Data was expressed as mean ± standard deviation (SD). The comparison between two groups was analyzed using independent sample t-test and one-way ANOVA followed by Bonferroni's post-hoc comparison tests was performed in multigroup comparisons. A *P* < 0.05 indicated a significant difference.

## 3. Results

### 3.1. Identification of AR Animal Model

**Figure 1:**
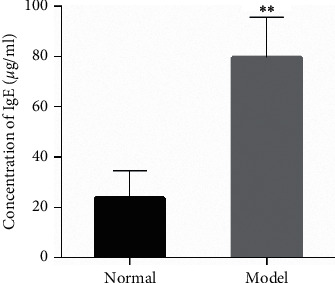
Concentration of serum IgE in AR guinea pigs by ELISA. Data were shown as mean ± SD (normal group, *n* = 10; model group, *n* = 18). ^*∗∗*^*P* < 0.01 vs. the normal group. AR: allergic rhinitis.

Twenty guinea pigs replicated the AR model by OVA sensitization, and one guinea pig died during the sensitization phase and the other one during the challenge phase. The behavioral rating scale after the last challenge showed that the AR model of 17 guinea pigs replicated successfully with a success rate of 85% (Table 2). The serum IgE detected by ELISA showed that the concentration of IgE of 18 guinea pigs in the model group was higher than that in the normal group, with a significant statistical difference (*P* < 0.01) (Figure 1).

### 3.2. Morphological Observation of Fibroblasts

**Figure 2:**
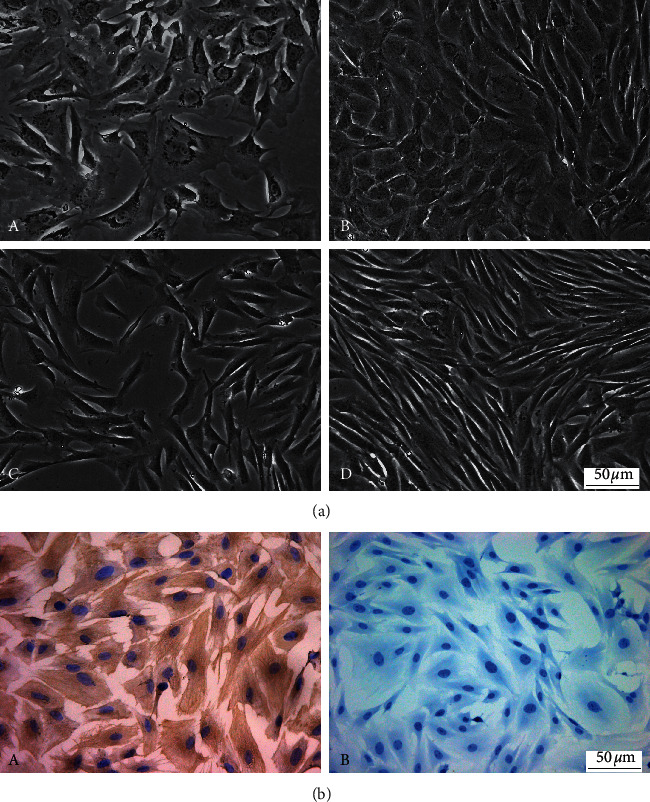
Morphology characteristics and identification of fibroblasts. Magnification, ×200; scale bar = 50 *µ*m. (a) Morphology characteristics of nasal mucosal fibroblasts of guinea pigs: (A) primary fibroblasts cultured for three days, (B) primary fibroblasts cultured for eight days, (C) F3 generation fibroblasts cultured for three days, and (D) F3 generation fibroblasts cultured for five days. (b) Identification of fibroblasts by immunocytochemical staining of vimentin: (A) vimentin is positively expressed in fibroblast cytoplasm; (B) no expression of vimentin was observed in the negative control group. The nuclei were counterstained with hematoxylin (blue).

After being cultured for three days in primary nasal mucosa fibroblasts, the morphology of the cells was diversified under an inverted phase contrast microscope. Most of the cells were spindle-shaped and polygonal, with large cell bodies, oval nucleus, and many granules in the cytoplasm, and they were scattered in clusters. After being cultured for 7∼8 days, the cells merged into flakes. Under the microscope, different morphological cells were mixed and grown, which were mainly fusiform and closely arranged flattened cells. When the cells were passaged and purified to the F3 generation, the cells grew vigorously, and the morphology was uniform, mostly in long fusiform and distributed evenly. When the density was high, they were in a pattern resembling a shoal of fish and in a radial-shaped form (Figure 2(a)).

### 3.3. Identification of Fibroblasts by Immunocytochemistry

Immunocytochemical staining of F3 generation fibroblasts showed that the cytoplasms were brownish yellow, the vimentin expression was positive, and the cytoplasms of the negative control group were not stained (Figure 2(b)).

### 3.4. Effects of TGF-*β*1, XGND, and Rhinocort on the Viabilities of Fibroblasts

**Figure 3:**
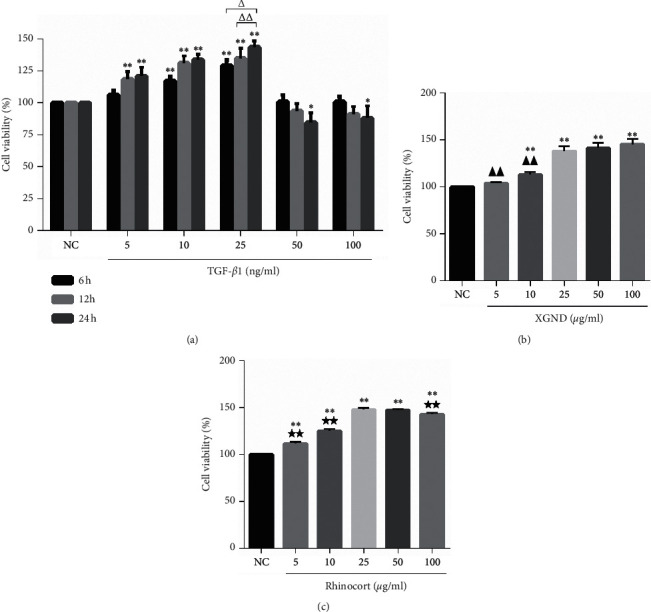
Effect of different concentrations of TGF-*β*1, XGND, and Rhinocort on viabilities of fibroblasts. Screening the optimal concentration of TGF-*β*1, XGND, and Rhinocort using CCK-8 assay. Data were shown as mean ± SD from three independent experiments. ^*∗*^*P* < 0.05 and ^*∗∗*^*P* < 0.01 vs. the NC group. ^△^*P* < 0.05 and ^△△^*P* < 0.01 vs. cells treated with 25 ng/ml TGF-*β*1 for 24 h. ^▲▲^*P* < 0.01 vs. XGND 25 *μ*g/ml for 24 h. ^★★^*P* < 0.01 vs. Rhinocort 25 *μ*g/ml for 24 h. TGF-*β*1: transforming growth factor-*β*1; XGND: Xingbi gel nasal drops; NC: negative control.

To investigate the effects of TGF-*β*1, XGND, and Rhinocort on the viabilities of nasal mucosa fibroblasts of AR guinea pigs, a CCK-8 assay was performed. As shown in Figure 3(a), the fibroblasts were treated with different concentrations of TGF-*β*1 for 6 h, 12 h, and 24 h, respectively. Compared with negative control (NC) group, the cells viabilities increased significantly with the concentration- and time-dependence after the treatment with TGF-*β*1 5 ng–25 ng/ml (treatment with 5 ng/ml TGF-*β*1 for 12 h and 24 h, all *P* < 0.01; treatment with 10 ng/ml and 25 ng/ml TGF-*β*1 for 6 h, 12 h, and 24 h, respectively, all *P* < 0.01); after 6 h and 12 h treatment of TGF-*β*1 50 ng/ml and 100 ng/ml, there was no significant change in cells viabilities; and after treatment for 24 h, the cells viabilities decreased significantly (both *P* < 0.05). The results showed that the cells viabilities increased most significantly after treatment with 25 ng/ml TGF-*β*1, and the cell viability increased to 129.356 ± 4.354% after 6 h, 134.810 ± 8.047% after 12 h, and 143.824 ± 4.773% after 24 h. Compared with treatment for 6 h and 12 h, treatment for 24 h showed significant differences (*P* < 0.01 and *P* < 0.05, resp.). In addition, the fibroblasts were cultured in different concentrations of XGND and Rhinocort for 24 h. As shown in Figures 3(b) and 3(c), compared with NC group, the cells viabilities increased significantly after treatment with XGND and Rhinocort for 24 h (treatment with 10 *μ*g/ml∼100 *μ*g/ml XGND, all *P* < 0.01; treatment with 5 *μ*g/ml∼100 *μ*g/ml Rhinocort, all *P* < 0.01), indicating that both XGND and Rhinocort can induce the proliferation of fibroblasts. The cell viabilities increased to 138.209 ± 4.892% and 147.760 ± 1.856% after treatment with 25 *μ*g/ml XGND and Rhinocort, respectively. Compared with the 25 *μ*g/ml concentration group, there was no significant difference in cell viabilities after treatment with 50 *μ*g/ml and 100 *μ*g/ml XGND and 50 *μ*g/ml Rhinocort, while the cell viability of 100 *μ*g/ml Rhinocort group was lower than 25 *μ*g/ml Rhinocort group (*P* < 0.01). Therefore, treatments with 25 ng/ml TGF-*β*1, 25 *μ*g/ml XGND, and 25 *μ*g/ml Rhinocort for 24 h were chosen as the best conditions in the nasal mucosa fibroblasts of AR guinea pigs.

### 3.5. Real-Time PCR

**Figure 4:**
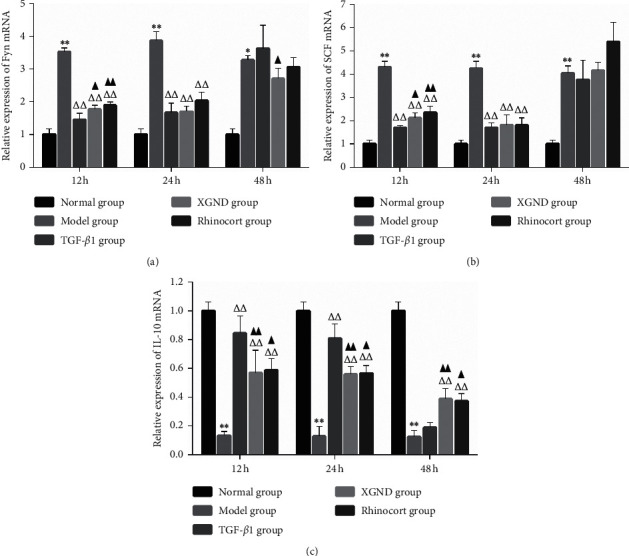
Effect of XGND on the expression of Fyn, SCF, and IL-10 mRNA in AR fibroblasts. The relative mRNA expression levels of (a) Fyn, (b) SCF, and (c) IL-10 were detected by real-time PCR method after fibroblasts were treated with XGND for 12 h, 24 h, and 48 h Data were shown as mean ± SD from three independent experiments. ^*∗*^*P* < 0.05 and ^*∗∗*^*P* < 0.01 vs. the normal group. ^△^*P* < 0.05 and ^△△^*P* < 0.01 vs. the model group. ^▲^*P* < 0.05 and ^▲▲^*P* < 0.01 vs. the TGF-*β*1 group. AR: allergic rhinitis; SCF: stem cell factor; IL-10: interleukin-10; TGF-*β*1: transforming growth factor-*β*1; XGND: Xingbi gel nasal drops.

As shown in Figure 4, the expression levels of Fyn and SCF mRNA in the model group were significantly higher than those in the normal group (*P* < 0.01), while IL-10 mRNA in the model group were significantly lower than that in the normal group (*P* < 0.01). Compared with the model group, the expression levels of Fyn and SCF mRNA were remarkably decreased and IL-10 mRNA were remarkably increased in the three treated groups after treatment for 12 h and 24 h (all *P* < 0.01), and IL-10 mRNA in XGND group and Rhinocort group were both significantly increased after treatment for 48 h (both *P* < 0.01). Compared with the TGF-*β*1 group at the same time point, the expression levels of Fyn and SCF mRNA in the XGND group were increased after treatment for 12 h = (both *P* < 0.05), but there was no significant difference after treatment for 24 h (both *P* > 0.05), while IL-10 mRNA decreased observably after treatment for 12 h and 24 h (both *P* < 0.01). After treatment for 48 h, the expression level of Fyn mRNA in XGND group was remarkably lower than that in TGF-*β*1 group (*P* < 0.05), and IL-10 mRNA was remarkably higher than that in TGF-*β*1 group (*P* < 0.01). Compared with Rhinocort group at the same time point, there was no significant difference in the mRNA expression of each gene in XGND group (all *P* > 0.05).

### 3.6. Western Blot Analysis

**Figure 5:**
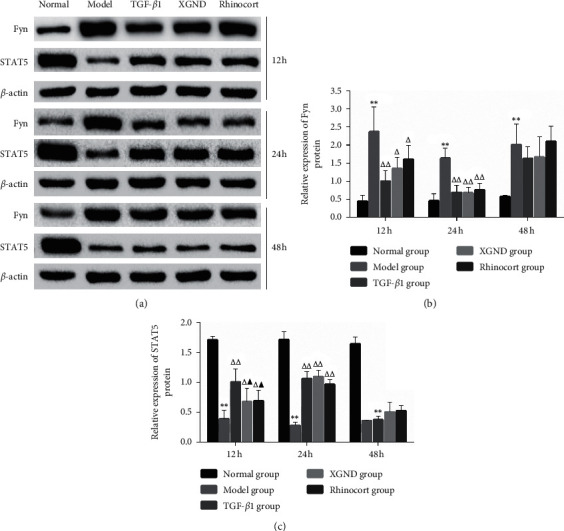
Effect of XGND on the expression of Fyn-STAT5 pathway protein in AR fibroblasts. The relative protein expression levels of Fyn and STAT5 were analyzed by western blot method after fibroblasts were treated with XGND for 12 h, 24 h, and 48 h. (a) The protein images of Fyn and STAT5 in different groups by ECL chemiluminescence. *β*-actin was an internal control. (b) The protein gray value analysis result of Fyn. (c) The protein gray value analysis result of STAT5. Data were shown as mean ± SD from three independent experiments. ^*∗*^*P* < 0.05 and ^*∗∗*^*P* < 0.01 vs. the normal group. ^△^*P* < 0.05 and ^△△^*P* < 0.01 vs. the model group. ^▲^*P* < 0.05 and ^▲▲^*P* < 0.01 vs. the TGF-*β*1 group. AR: allergic rhinitis; STAT5: signal transduction and activator of transcription 5; TGF-*β*1: transforming growth factor-*β*1; XGND: Xingbi gel nasal drops.

As shown in Figure 5, compared with the normal group at the same time point, the expression level of Fyn protein in the model group increased significantly (*P* < 0.01), while STAT5 protein decreased significantly (*P* < 0.01). Compared with the model group at the same time point, the protein level of Fyn decreased remarkably and STAT5 increased remarkably in the three treated groups after treatment for 12 h and 24 h, respectively (*P* < 0.01, *P* < 0.05); after treatment for 48 h, the protein levels of Fyn and STAT5 were not significantly different from that of the model group (both *P* > 0.05). Compared with TGF-*β*1 group at the same time point, the protein levels of Fyn in XGND group were not significantly different after treatment for 12 h, 24 h, and 48 h (all *P* > 0.05), while STAT5 protein in XGND group was observably decreased after treatment for12 h (*P* < 0.05). However, there were not significant differences after treatment for 24 h and 48 h (both *P* > 0.05). Compared with the Rhinocort group at the same time point, there was no significant difference in the levels of each protein in the XGND group (all *P* > 0.05).

## 4. Discussion

Fibroblasts of nasal mucosa can participate in inflammatory reactions and damage repair of various nasal diseases by secreting a variety of cytokines and chemokines [9, 10]. In the current study, XGND was used to treat the nasal mucosa fibroblasts of AR guinea pigs, and TGF-*β*1 and Rhinocort were used as positive controls to verify the regulatory effects of XGND on AR and Fyn-STAT5 signaling pathway. The results showed that the mRNA and protein levels of Fyn and SCF were highly expressed in AR nasal mucosa fibroblasts, while IL-10 and STAT5 were lowly expressed, suggesting that Fyn, STAT5, IL-10, and SCF were all associated with AR.

Previous studies on the interaction between Fyn and STAT5 have focused on the regulation of various cancers and leukemias [11–13]. Our study is the first to investigate the relationship between Fyn/STAT5 and allergic rhinitis. Fyn, a nonreceptor tyrosine kinase in cells, is an important member of the Src kinase family. It plays a major role in the positive regulation of Fc*ε*RI-mediated degranulation of mast cells and is located in the upstream stage of activation and degranulation of mast cells [14]. STAT5 is an important member of the STAT family and plays a key role in the development and survival of mast cells, which mediates allergic reactions activated by mast cells. The STAT5 signal transduction pathway can activate or inhibit nuclear transcription by phosphorylating tyrosine residues of STAT5 via activation of JAK2 kinase. In addition, STAT5 is involved in the functional decision-making of plasma cells and memory cells and plays an important role in self-renewal of B cells and inhibition of plasma cell differentiation. According to the regulation of Fyn and STAT5 on mast cell activation and IgE receptors, Pullen et al. found that Fyn could induce phosphorylated STAT5 to approach the proximal FC*ε*RI in an IgE-dependent and non-IL-3/SCF-dependent manner in mast cells, which causes STAT5 and Fyn to form an immune complex and deposit in mast cells that have not yet been activated [15]. When Fyn-STAT5 signaling pathway is activated in mast cells, it will transduce information into the nucleus, activate the signal transduction in the nucleus, and ultimately lead to degranulation of mast cells and a series of allergic reactions [5].

In allergic diseases, TGF-*β*1 has the function of inhibiting mast cells [16]. IL-10 is an important cytokine that regulates airway inflammation, which has negative immunoregulatory effects such as reducing T cell response, enhancing B cell function, and inhibiting the synthesis of proinflammatory cytokines [17]. Adding exogenous IL-10 and TGF-*β*1 into mast cells can lead to a gradual decrease in the protein levels of Fyn and STAT5 [18]. SCF is an important hematopoietic regulatory factor, which can widely regulate the production of hematopoietic cells and immune cells.

In the current study, TGF-*β*1, XGND, and Rhinocort can induce the proliferation of the nasal mucosa fibroblasts of AR guinea pigs in a certain extent. After treatment with XGND for 12 h and 24 h, the mRNA and protein levels of Fyn and SCF were significantly lower than those of model group, and the expression of IL-10 gene and STAT5 protein was significantly increased, which was consistent with the regulatory trend of TGF-*β*1 group and Rhinocort group, but the 12 h regulation of XGND treatment is weaker than that of the TGF-*β*1 group. After treatment for 24 h, the upregulation of IL-10 mRNA in XGND group was inferior to that in TGF-*β*1 group, and other mRNAs and proteins were not significantly different from those in TGF-*β*1 group. After treatment for 48 h, the expression of Fyn, SCF, and STAT5 in XGND group was not significantly different from that in the model group, suggesting that the pharmacodynamic effect of XGND group had been weakened, and the regulation ability of XGND was consistent with that of TGF-*β*1 group. The gene level of IL-10 was still higher than that in model group, and the upregulation ability of XGND was better than that of TGF-*β*1 group.

In addition, compared with the expression of each target gene, there was no significant difference between XGND group and Rhinocort group at the same treatment time point, suggesting that the two drugs interfere with AR fibroblasts in a similar regulatory pathway. The results showed that XGND could reduce Fyn and SCF expression and hinder Fc*ε*RI receptor cross-linking with treatment for 24 h, inhibit proliferation and degranulation of mast cells, and reduce the release of histamine and various inflammatory cytokines. Meanwhile, it could upregulate STAT5 and antagonize IgE synthesis and correct Th1/Th2 immune imbalance, thereby inhibiting Fyn-STAT5 signal transduction pathway and relieving AR fibroblasts immune response. In addition, XGND could significantly increase IL-10 and inhibit the production of various proinflammatory factors, thereby reducing airway inflammatory response and regulating the body's immune function.

XGND is composed of *Radix cynanchi Paniculati* (Pycnostelma paniculatum K. Schum), *Periostracum Cicadae* (cicada slough), *Calculus bovis Artifactus* (artificial bezoar), and *Borneolum Syntheticum* (borneol). The main effective components of *Radix cynanchi Paniculati* are paeonol and saponins, which have anti-inflammatory, bacteriostatic, analgesic and antipyretic, and antiallergic effects, and regulate the immune function. *Periostracum Cicadae* exuviation contains a large number of chitin, chitosan, various amino acids, and proteins. It also has sedation, antipyretic, and immunosuppression effects. Artificial bezoar is made by artificially extracting bile acid, cholesterol, bilirubin, inorganic salt, and the like from bovine bile or pig bile by reference to the active ingredient of natural bezoar, which can enhance immunity and antioxidation by regulating mononuclear phagocytic system and B lymphocytes. *Borneolum Syntheticum* can open blood-brain barrier, promote drug absorption and penetration, and improve bioavailability. Therefore, it is often used as a penetration enhancer in various external preparations. Alternatively, with liposome as the medium, XGND can prolong the time of drugs acting on nasal mucosa and effectively improve the bioavailability while reducing the dosage of drugs.

The previous clinical studies showed that XGND can significantly reduce the levels of inflammatory factors such as serum IgE, Fc*ε*RI, TSLP, and IL-4, inhibit the release of inflammatory mediators, decrease the inflammatory cell aggregation in nasal mucosa, and reduce the inflammatory reaction of the nose. Besides, compared with intranasal corticosteroids, the efficacy of XGND is more stable and lasting in the Symptoms and Signs Scale and the Quality of Life Questionnaire score. In vivo studies by Nan et al. and Chen et al. confirmed that XGND can significantly reduce the mRNA and proteins expression levels of NF-*κ*B and NF-*κ*B p65 in nasal mucosa of AR guinea pigs and reduce the concentrations of inflammatory factors such as IL-5, GM-CSF, and CCL-1 in serum and nasal lavage fluid, suggesting that XGND can also restrain the allergic reaction by inhibiting the activation and nuclear translocation of NF-*κ*B and weakening the biological effects of IL-5, GM-CSF, CCL1, and other inflammatory factors [19, 20]. Further studies by Ai et al and Wang et al found that XGND can significantly reduce the concentration of IgE, IL-4, IL-5, LTE4, and tryptase in the serum and decrease eosinophilia in the nasal mucosa of AR guinea pigs and rats, by downregulating PLCE1-PKC-NF-*κ*B signaling pathway, correcting Th1/Th2 immune imbalance, and thereby inhibiting the release of inflammatory mediators, to alleviate allergic symptoms in AR guinea pigs and rats [21, 22].

## 5. Conclusions

In conclusion, XGND may interfere with the Fyn-STAT5 pathway, thereby inhibiting proliferation and degranulation of mast cells, correcting Th1/Th2 immune imbalance, and then alleviating the immune response of AR fibroblasts. One of the limitations of our study is the choice of animal species. Guinea pigs are the most classic and sensitive experimental animals for the study of allergic diseases. However, as there are a few ingrained strains of guinea pigs and some gene sequences are still unclear, it is difficult to carry out more in-depth mechanism research. In order to clarify the role and safety of XGND in AR therapy, further studies are needed.

## Figures and Tables

**Table 1:** Primer sequences for real-time PCR.

Primer	Sequence	Length of product (bp)
GAPDH	F: 5′—ACCTAATGTGTCGGTTGTGGAT—3′	169
	R: 5′—GGAAGAATGGCTGTCACTGTTG—3′	

Fyn	F: 5′—GGTGTTTCGCTGAAGTGTGGCT—3′	271
	R: 5′—ATGTCCACGAGATTGGGTAACTTC—3′	

IL-10	F: 5′—GCACAAGTCATTGCTGGAGGAT—3′	170
	R: 5′—TACGCAGCCTGAGGGTCTTCA—3′	

SCF	F: 5′—GACATAGACCAGGCACACGCATA—3′	208
	R: 5′—TTGCCAGGGATGCCAAATGTC—3′	

**Table 2:** Behavioral rating scale of AR guinea pigs.

Times of challenge	Total score <5	Total score >5	Points	Remarks
1st	15	4	3.21 ± 1.39	1 died in the sensitization phase
2nd	11	8	4.58 ± 1.26	
3rd	6	13	5.47 ± 1.39	
4th	3	15	5.78 ± 1.31	1 died on the 4^th^ challenge
5th	1	17	6.44 ± 1.15	1 score <5

## Data Availability

The data used to support the findings of this study are available from the corresponding author upon reasonable request.
